# A Case of Rabies

**Published:** 1881-10

**Authors:** William Henry Porter


### CASE DEPARTMENT.

### I.—A CASE OF RABIES.

REPORTED BY WILLIAM HENRY PORTER, M.D., V.S.

A dog named Mina, and owned by Mr. Walter H. Beebe, of New York City, was a valuable English setter, aged four years and three months. This animal was bitten by a common cur, which was supposed to be mad, on June 30th, 1881, at Morris Plains, New Jersey. Bluebelle, another fine bitch, also owned by Mr. B., was bitten the same evening. It is an open question as to which dog was first attacked, but both of them were severely bitten many times, about the mouth and shoulders; Mina, in one place, quite through the upper lip. After this the cur disappeared, and what became of him was never positively known. Bluebelle was expected to have whelped the following day, but this she failed to do, and has not since.

July 5th Mr. B. saw both dogs, and found them in fine condition, the injuries giving no trouble, and his impression was that the wounds were quite healed, and they appeared to be feeling perfectly well; Mina quiet, and not especially excitable. They remained well, and the wounds gave no trouble until July 18th, when Mina refused to eat, and continued to fast up to July 21st, when the owner was first notified of the fact.

Mr. B. talked of bringing the animal to the Columbia Veterinary College Hospital for treatment, and in case of death, for the benefit to science from an early necropsy in a well-watched case.

On Saturday, July 23d, the owner went to Morris Plains, with the above stated purpose in view, his keeper, Mr. Parrol, having thought the dog too excitable to handle alone. When Mr. B. arrived at the farm, however, the dog seemed quite manageable, and anything but mad, and jumped up to meet him as usual. The wounds about the mouth and chest were not extensively inflamed, but the lips and tongue were swollen, the latter being very red, and covered with a thick coating; the margins of the tongue, however, were quite red, but free from sordies. The dog had often been boxed for transportation, but never muzzled, to his knowledge. Just before attempting to muzzle her, she had a severe attack of dry retching, but without expelling anything from the mouth. The attack finally terminated in a convulsive cough, after which the coating on the tongue was less marked. After this a muzzle was carefully slipped over the nose and fastened, an operation which she bore quite well; but when about to be put into the box the muzzle seemed to come in contact with the inflamed lips, which almost immediately threw her into a frenzy, which was frightful to behold. She was, however, got into the box, which was bound together with strong cord, to prevent any possibility of her breaking out. During this frenzy her mouth became badly cut from biting at the wire muzzle, and both blood and froth, probably saliva, flew in all directions from her mouth, and in considerable quantities. After about ten minutes of this violent struggling she became, apparently, exhausted, a condition which was thought in part, however, to be due to the safety cord about her neck, that partially strangled her. She fell down in the box, and after a few gasps was dead, her teeth firmly clasping a part of the muzzle.

It might be well to mention, in connection with the above history, that Mina was removed from her kennel among some other dogs, and placed alone in a part of the barn where there was no floor. Some straw, however, was furnished her as a bed, but she refused to lie upon it, preferring to lie upon the damp earth.

Unfortunately, was unable to make the necropsy until forty-eight hours after death. Immediately after death the dog was boxed and buried in a damp, shady place on the farm, and not exhumed until the time of the necropsy. The animal was well supplied with adipose tissue. There was only a slight amount of decomposition at this time. The lips and cheeks were very much swollen, and the surrounding areolar tissue filled with bloody serum. The whole neck, in fact, was swollen, and infiltrated in the same way.

*Thoracic Cavity.*—The pericardium contained a small amount of bloody serum. The right cavities were distended with blood and coagulum; the left cavities nearly empty. Microscopically the heart fibres were found to be normal. The lungs were very deeply congested, very dark red in color, but otherwise they appeared normal.

*Abdominal Cavity.*—The portal vein, its branches, and their tributaries were distended with blood. The spleen appeared normal. The kidneys were deeply congested, and microscopically the epithelium was very granular, but this may have been in part due to the time which had elapsed between death and the necropsy. The liver was deeply congested; in fact all the internal organs were intensely congested. The cranial cavity and spinal canal were laid open. The meninges of the brain and cord were not congested, but, on the contrary, anæmic. The brain substance also was very pale. The brain was found too soft to remove with any prospect of preserving. The upper portion of the spinal cord was carefully removed, but it was impossible to harden it sufficiently to make any satisfactory section, therefrom.

The question as to the case being one of rabies is somewhat doubtful. But from the fact that the dog apparently died from asphyxia—which is considered as the immediate cause of death in rabies—I think even in absence of any further positive proof the case should be regarded as one of rabies, and the other dog bitten closely watched for six months at least.

This case, I think, will be of interest in connection with the pathology of rabies and hydrophobia, copied from Dr. T. E. Satterthwaite's article on the same.

---

We insert the following in connection with Dr. Porter's case. It represents the latest facts known regarding the subject, and will be found of much interest.—Ed.

### PATHOLOGICAL ANATOMY OF RABIES, BY DR. T. E. SATTERTHWAITE, IN SUPPLEMENT TO "ZIEMSEUS' CYCLOPEDIA OF THE PRACTICE OF MEDICINE."

---

Dr. R. W. Gowers has devoted careful attention to this subject, and while he disclaims having noticed any pathological changes that are distinctive of the disease, he has found a certain degree of uniformity in them. In the gray matter of the medulla and cord he observed great distention of the vessels. This condition was regarded as pathological, even after taking into consideration that death in hydrophobia is from asphyxia. The vascular distention was most abundant about the ganglionic bodies which lie beneath the floor of the fourth ventricle—a condition which is regarded by Dr. A. W. Foote as explaining the frequency of glycosuria in hydrophobia. In the region of the nuclei of the glossopharyngeal, and hypoglossal and pueumogastric nerves, the perivascular sheaths were most markedly engorged with blood. The greatest intensity of the pathological change coincides with the area that has been termed the respiratory centre. Dr. Gowers, however, had seen similar collections in chorea in a dog. He lays weight, however, upon the site of these changes in hydrophobia.

Though peculiar changes have been described in the follicular glands, tonsils, and remaining parts of the throat, Fahrer asserts that in some well-marked cases these have been absent.

Bollinger, however, gives a case which came under his observation at the Munich Hospital, in October, 1875. There was moderate reddening of the pharynx, with ecchymosis in the vocal cords; the lungs also were full of blood, the right lower lobe being in a state of splenization, and there was croupous bronchitis. Both of these latter lesions, however, were caused by the entrance of foreign bodies into the lungs—in this case milk and chloral—which accidentally deviated from its proper course while the patient was being artificially nourished.

Allbutt and Coates, according to Fahrer, describe changes in the nerve-cells and vessels, deposits of round corpuscles in the perivascular spaces, also granular degeneration, and even embolism, mostly about the respiratory centre.

Hammond has detailed the history of a case which he saw in June of 1874. The pathological appearances which were described in this case, and which concerned the nervous system, were briefly as follows :

In the cortical substance of the brain, an increase in size and number of the blood-vessels, with minute extravasations of blood. The first and second layers of cells had been replaced in large part by fatty matter. Between the two layers were anyloid corpuscles. The third layer was but little changed, and the others appeared not to have been involved. In the pons varolii, enlargement and thickening of vessels and extravasations were also seen. In sections made through the medulla, so as to include the nuclei of the hypoglossal and pneumogastric, there were also numerous extravasations.

Benedick also has lately described peculiar inflammatory changes in the brain of rabid dogs, and in the case of a man. The morbid appearances are classed as granular degeneration, miliary abscesses and hemorrhages.

N. Kalisvukoff has further described similar appearances, but neither of these alleged findings could be sustained by Dr. Augustus Forel. His studies, conducted by excellent methods, and as yet unpublished, were made upon the brain of a hydrophobic man and several rabid animals (*Bollinger*).

On the other hand, Drs. R. H. Fitz and Shattuck, of Boston, in concluding their history of a case which had been under most excellent observers, and where the post-mortem and subsequently the microscopic examinations were conducted with unusal care, make the following statement:

"In brief, then, the alterations were a diffuse cellular infiltration of the adventitia of the veins, venous injection and thrombosis, perivenous hemorrhages and miliary abscesses." The "hyaloid masses," which have been described, are, they state, very often met with in healthy brains and cords hardened in chromic acid and its compounds, and are, probably, the results of decomposition, independent of any pathological changes.

